# A structural connectivity convergence zone in the ventral and anterior temporal lobes: Data-driven evidence from structural imaging

**DOI:** 10.1016/j.cortex.2019.06.014

**Published:** 2019-11

**Authors:** Claude J. Bajada, Nelson J. Trujillo-Barreto, Geoff J.M. Parker, Lauren L. Cloutman, Matthew A. Lambon Ralph

**Affiliations:** a Division of Neuroscience & Experimental Psychology, School of Biological Sciences, The University of Manchester, UK; b Faculty of Medicine and Surgery, University of Malta, Malta; c MRC, Cognition and Brain Sciences Unit, The University of Cambridge, Cambridge, UK; d Bioxydyn Limited, Manchester, UK; e Centre for Medical Image Computing, Department of Computer Science, and Queen Square MS Centre, Department of Neuroinflammation, UCL Institute of Neurology, University College London, UK; f Department of Physiology and Biochemistry, Faculty of Medicine and Surgery, University of Malta, Malta

**Keywords:** Semantics, Connectivity, Anterior temporal lobe, Spectral reordering, Laplacian eigenmapping

## Abstract

The hub-and-spoke model of semantic cognition seeks to reconcile embodied views of a fully distributed semantic network with patient evidence, primarily from semantic dementia, who demonstrate modality-independent conceptual deficits associated with atrophy centred on the ventrolateral anterior temporal lobe. The proponents of this model have recently suggested that the temporal cortex is a graded representational space where concepts become less linked to a specific modality as they are processed farther away from primary and secondary sensory cortices and towards the ventral anterior temporal lobe.

To explore whether there is evidence that the connectivity patterns of the temporal lobe converge in its ventral anterior end the current study uses three dimensional Laplacian eigenmapping, a technique that allows visualisation of similarity in a low dimensional space. In this space similarity is encoded in terms of distances between data points.

We found that the ventral and anterior temporal lobe is in a unique position of being at the centre of mass of the data points within the connective similarity space. This can be interpreted as the area where the connectivity profiles of all other temporal cortex voxels converge.

This study is the first to explicitly investigate the pattern of connectivity and thus provides the missing link in the evidence that the ventral anterior temporal lobe can be considered a multi-modal graded hub.

## 1 Introduction

Semantic memory is often described as ‘knowing what you know’. It is usually contrasted with episodic memory which can be thought of ‘knowing when you know’. For example, knowing that you ate a delicious croissant for breakfast yesterday is an example of episodic memory (when). In contrast, knowing that a croissant is a delicious French pastry rather than a venomous bog dwelling amphibian, is an example of semantic memory (what). The temporal lobe has been implicated in both of these cognitive processes, with episodic and semantic memory associated with different anatomical sub-regions. The medial temporal lobe and hippocampus have famously been associated with episodic memory (Scoville & Milner, 1957). There are two prominent brain-based models of the semantic system. The first ‘distributed-only’ model posits that semantics emerges as a feature of a widely distributed network of primary cortices and their association areas (Martin, 2007). Within this model, modality-specific information types (e.g., visual, auditory) are stored within their respective primary cortical regions (spokes), with interconnections between them producing a full semantic description of a concept. The second, ‘hub-and-spoke’ model extends this idea to propose the existence of a central cortical hub which receives and integrates this distributed modality-specific information from the spokes to derive transmodal, generalizable concepts (Lambon Ralph, 2014, Lambon Ralph et al., 2017, Patterson et al., 2007, Rogers et al., 2004).

Parts of the ventrolateral anterior temporal lobe have been shown to be crucial for the processing of semantic information (Lambon Ralph et al., 2017). Evidence for the importance of the ATL in general initially arose from semantic dementia, a neurodegenerative condition characterised by the progressive loss of conceptual information, with the centre of neural degeneration and hypometabolism occurring primarily around the anterior temporal lobes (ATL) in both hemispheres (Galton et al., 2001, Mion et al., 2010, Mummery et al., 2000, Nestor et al., 2006). The condition gave rise to a body of research that implicated the ATLs as an area critical to the processing of conceptual knowledge (Bozeat et al., 2000, Ding et al., 2016, Hodges et al., 1992). With the advent of advanced neuroimaging and neurostimulation methods, such as functional magnetic resonance imaging (fMRI), transcranial magnetic stimulation (TMS) and sub-dural electrode studies, a converging body of evidence has implicated the bilateral ventral ATLs as the key regions involved in multi-modal conceptual integration (Abel et al., 2015, Binney et al., 2010, Chen et al., 2017, Noonan et al., 2013, Pobric et al., 2007, Rice et al., 2015, Rice et al., 2015, Shimotake et al., 2015, Visser et al., 2010). Indeed, the original implementation of the hub-and-spoke model considered the bilateral ATLs as the core region where convergence and integration of modality-specific information took place (Rogers et al., 2004). However, recent accounts of this model have suggested that semantic specialisation is likely to be graded where, at one extreme, a region will process modality-specific semantics (e.g., visual, auditory etc.), with this information gradually converging onto ATL regions that activate independent of input modality (the fully multi-modal hub area) (Binney et al., 2012, Lambon Ralph et al., 2017, Plaut, 2002, Rice et al., 2015).

While evidence has accumulated for the role of the ventral ATLs in integrative multi-modal semantic processing, the underlying network of white matter connections via which this integration takes place has been less explored. It would seem likely that such an integrative system would require a neural architecture that connects primary and secondary sensory regions to the tertiary ‘hub’, either directly, with integration occurring only at the endpoint within the hub, or more gradually, with a graded convergence of connectivity and information. Evidence for this former system was found in a probabilistic tractography study conducted by (Binney et al., 2012). This study found that long range connectivity from primary sensory areas showed a pattern of gradual convergence within the ATLs, and that an area within the ventral ATL region exhibited a high degree of intralobar connectivity. The study, however, focused on the white matter connectivity of specific regions of interest and a map documenting the gradations in connectivity across the temporal cortex was not within the scope of that work. As such, the question regarding the pattern and extent of convergent connectivity within temporal lobe is still currently unclear. Furthermore, it is unclear as to whether or how the connectivity of the ATL is distinct, suggesting a specialised role, or in fact similar to other regions of the lobe, such as the medial temporal lobe and other parts of the lateral cortex.

A technique which has emerged in the neuroscience literature, aimed at probing the underlying shifts in cortical connectivity, is Laplacian eigenmapping or spectral embedding (Belkin and Niyogi, 2002, Belkin and Niyogi, 2003). This technique has its roots in graph theory (Barnard et al., 1993, Barnard Stephen et al., 1995, Fiedler, 1973) and has had a long history in the machine learning literature (Ng et al., 2002, Shi and Malik, 1997, Shi and Malik, 2000, Von Luxburg, 2007). In neuroscience, the technique has successfully been used to cluster neural regions (Craddock et al., 2012, Eickhoff et al., 2015, Johansen-Berg et al., 2004), as well as, more recently, to extract the primary gradients of structural and functional connectivity across several cortical regions, such as the insula, the motor cortex and temporal lobe (Cerliani et al., 2012, Haak et al., 2016, p. 1602; Jackson, Bajada, Rice, Cloutman, & Lambon Ralph, 2017) and of the cerebellum (Guell, Schmahmann, Gabrieli, & Ghosh, 2018). The technique involves extracting a region of interest and computing similarities between the connectivity profiles of each voxel in the region. These similarities are then reduced to a lower dimensional space whereby each point represents a cortical voxel, and its closeness to other points in space represents its similarity in connectivity. Consequently, a map can be generated which delineates the patterns of connective similarity across regions, and the gradual and graded transitions in connective patterns (Margulies et al., 2016).

In the current work, this data driven approach was used to map the patterns of connective architecture across the temporal lobe. An organisational structure was revealed which is consistent with a pattern of convergence from primary sensory areas to the ventral ATL. The maps obtained are strikingly similar to the graded convergence of structure and function toward the ventral ATL proposed by (Binney et al., 2012) and (Rice, Hoffman, et al., 2015). The results provide evidence that the ventral ATL is a graded hub in the centre of the connectivity space.

## 2 Methods

### 2.1 Participants

Twenty-four healthy participants (11 females) were recruited, they explicitly had no history of neurological or psychological disorders. The mean age was 25.9 years with a range between 19 and 47 years. Handedness was measured on the Edinburgh Handedness Inventory (Oldfield, 1971) and only right handed participants were included in the study. All participants gave their informed consent and the research was approved by the local ethics committee (The University of Manchester). This study uses the same population sample and pre-processing steps as a previously published study (Bajada et al., 2017). We opted to use local data as opposed to switching to the publicly available, high quality Human Connectome Project data in order to provide continuity and results that are comparable to our previous work. We present a short summary of the pipeline below where we report all measures and manipulations.

Sample size was determined by the total amount of participants with DWI available in the local database. The exclusion criteria (no history of neurological or psychological disorders) were established prior to data collection. No data was excluded from the available cohort.

### 2.2 Image acquisition and pre-processing

All images were acquired on a 3 T Philips Achieva scanner (Philips Healthcare, Best, The Netherlands) using an 8 element SENSE head coil. Pulsed gradient spin echo echo-planar sequence diffusion-weighted images (DWI) were acquired with TE = 59 ms, TR ≈ 11,884 ms [cardiac gated was performed either by using a peripheral pulse monitor on each participant's index finger (n = 21), or by using electrocardiography (n = 3)], Gmax = 62 mT/m, half scan factor = .679, 112 × 112 image matrix reconstructed to 128 × 128 using zero padding, reconstructed in-plane voxel resolution 1.875 × 1.875 mm^2^, slice thickness 2.1 mm, 60 contiguous slices, 61 non-collinear diffusion sensitization directions at b = 1200 sec/mm^2^ (*Δ* = 29.8 ms, *δ* = 13.1 ms), 1 at b = 0 sec/mm^2^, SENSE acceleration factor = 2.5. Correction for susceptibility-related image distortions was performed using the approach outlined in (Embleton, Haroon, Morris, Ralph, & Parker, 2010), which involved the acquisition of two volumes with inversed phase encode directions (left-right) for each diffusion gradient direction. A T_2_-weighted turbo spin echo scan (in-plane voxel resolution of .94 × .94 mm^2^, slice thickness 2.1 mm) was obtained for a qualitative indication of distortion correction accuracy. Finally, a high resolution T_1_-weighted image was acquired for each participant using a 3D Turbo Field Echo inversion recovery scan (TR ≈ 2000 ms, TE = 3.9 ms, TI = 1150 ms, flip angle 8°, 256 × 205 image matrix reconstructed to 256 × 256, reconstructed in-plane voxel resolution .938 mm × .938 mm, slice thickness .9 mm, 160 slices, SENSE factor = 2.5).

The T1w image was co-registered to the diffusion images for visualisation and mask generation. A white matter mask (for tractography seed mask generation) and a cerebrospinal fluid mask (for a termination mask) were also generated from the co-registered T1w image using FSL's FAST algorithm.

### 2.3 Tractography

Tractography was performed using the probabilistic index of connectivity (PICo) algorithm (Parker and Alexander, 2005, Parker et al., 2003). The algorithm samples voxel-wise diffusion probability distribution functions (PDFs), generated via the constrained spherical deconvolution (Tournier, Calamante, & Connelly, 2007) and model-based residual bootstrapping method (Haroon et al., 2009, Haroon et al., 2009, Jeurissen et al., 2011).

To generate the temporal lobe region of interest (ROI) for tracking, a temporal mask was first defined in MNI space for both hemispheres using the MNI structural atlas within the FSL software package (Mazziotta et al., 2001). The mask was then co-registered to each participant's native diffusion space. To ensure that only voxels within the temporal lobe were included, the masks were manually reviewed in native space, and any voxels outside the temporal region removed. The final temporal ROI for tracking consisted of only those seed voxels at the interface between the grey matter and white matter. To generate this, a white matter mask was used to delineate those voxels at the grey–white interface (GWI), which were then extracted via an in-house MATLAB script (Bajada et al., 2017, The MathWorks, 2012).

For each voxel within a participant's ROI, unconstrained probabilistic tracking was performed by propagating 10,000 streamlines from the GWI to the rest of the brain. The parameters for the tracking included a step size of .5 mm and a curvature threshold of 180° over a voxel. Streamlines were terminated if they exceeded a path length of 500 mm or hit a cerebrospinal fluid (CSF) termination mask. A count of streamlines reaching every brain voxel was recorded, generating a connectivity profile for each temporal ROI seed voxel.

The connectivity profiles for each individual participant were transformed to a common template space using SPM's DARTEL (Ashburner, 2007). A GWI for the template space was also extracted using the script mentioned above. Every individual's connectivity profile was mapped onto a voxel on the group GWI template that was the nearest neighbour of that profiles seed voxel. This provided a correspondence of seed voxels and connectivity profiles across participants. The participants' connectivity profiles were then thresholded at .05% of the maximum value to remove very low level noise, and subsequently binarized (Devlin et al., 2006). The resulting images were down-sampled to half their size per dimension in order to reduce memory consumption, and the resulting profiles were averaged across participants to generate a group connectivity profile for each seed voxel.

### 2.4 Spectral embedding, laplacian eigenmapping and their visualisation

The group connectivity profile for each seed voxel consisted of a map of every cortical voxel within the whole brain connected with that seed. To examine the similarity between the connectivity profiles of the temporal seed voxels, the connectivity profiles were first transformed into a 1 × *m* row vector, where the columns (*m*) represented every point in the brain. A pairwise similarity of every seed voxel's connectivity profile was computed by calculating the cosine of the angle between each connectivity profile vector.

This generated an *n* x *n* symmetric similarity matrix where *n* = number of seed voxels in the temporal lobe's group GWI template. The similarity matrix was treated as a weighted graph adjacency matrix (A). The Laplacian of the adjacency matrix was computed by subtracting the adjacency matrix from the degree matrix (D):

(1) L=D−A

The eigenvectors associated with the three smallest nonzero generalised eigenvalues of L and D were computed by solving the generalised eigenvalue problem:

(2) L*X=D*X*Λ

where L is the Laplacian, D is the degree matrix, X is the matrix of generalised eigenvectors and Λ is a diagonal matrix of generalised eigenvalues. This was done in accordance with the (Shi & Malik, 2000) algorithm (see also (Von Luxburg, 2007) for a tutorial explanation).

The smallest eigenvalue of the Laplacian is always zero. If the zero eigenvalue has a multiplicity of greater than one, this means that the graph has more than one connected component. Clear clusters are denoted by small initial eigenvalues (very close to zero) followed by a large gap in the spectrum of eigenvalues between the initial eigenvalues and the rest of the eigenvalues.

The eigenvectors associated with the three smallest non-zero eigenvalues (the embedding eigenvectors) were plotted in a three-dimensional similarity space. This warps each temporal seed voxel into a three-dimensional space where every voxel is positioned according to their connective similarity to each other (see supplementary animation). Only three dimensions were selected for the following reasons. The largest gap within the eigenvalue spectrum is between the first and the second, this implies that the greatest dissimilarity lies within the first eigenvector (eigenmap) due to this, adding more than three eigenvectors would only add nuanced information to the visualisation. Estimation of dimension number is a difficult and often heuristic procedure. While this study opted for a heuristic approach, statistical approaches are being developed and may become standard as these techniques develop (Karolis, Corbetta, & Thiebaut de Schotten, 2018).

In order to produce a graded mapping from the similarity space to brain space, each voxel's coordinates in the similarity space were rescaled between 0 and 1 and used as an assigned to red, green and blue colour channels represented by the matrix RGB:

(3) $$\text{RGB}=\frac{X-1*X_{min}}{X_{max}-X_{min}}$$

where the three columns of the matrix *X* contain the embedding eigenvectors so that each row of *X* contains the coordinates of (or corresponding to) each seed voxel in the 3-dimensional similarity space; $X_{min}$ and $X_{max}$ denote respectively the minimum and maximum value in the matrix *X* and matrix **1** is a matrix of ones of the same size as X. The RGB colouring acts as a visualisation tool to aid the mapping of each voxel from one space (similarity space) to another (anatomic space) to allow a voxel to move in space but retain knowledge of how a voxel maps from one space to the other (see supplementary material for an animation of the mapping between similarity and anatomic spaces). The raw eigenvectors are susceptible to outlier voxels and these heavily restrict the RGB space that can be used. As such, the ‘raw’ eigenvectors were also modified by employing the rank order of the three smallest non-zero eigenvectors and represented in RGB as above to enhance the visualisation of the gradients (transition between ‘raw’ and ‘ranked’ RGB can be visualised in the supplementary material).

The distances between the data points in the ‘raw’ connectivity space are proportional to the similarity between the connectivity of the voxels. Hence, points that are close together in the similarity space have similar connectivity profiles. In order to highlight regions of the cortex that are maximally similar to all other areas, the centre of mass of the data in the similarity space was calculated. The Euclidean distance between all coordinates in the similarity space and the centre of mass was computed and the values that were within the closest 10th percentile were retained and projected back on both the connectivity space and on the cortex (see Fig. 2, Fig. 3A).

**Fig. 1:**
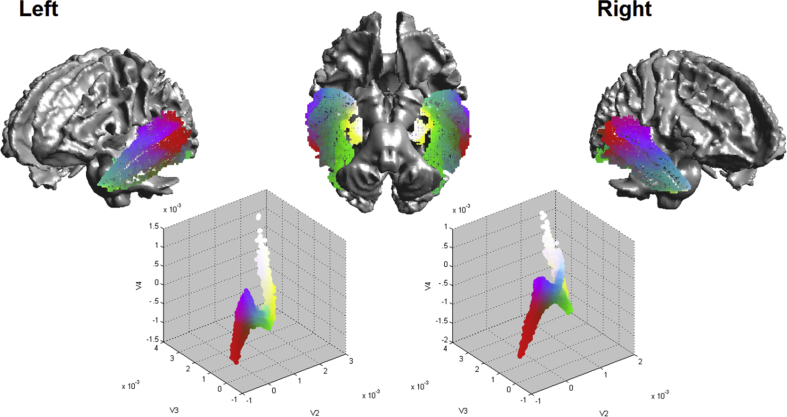
The bottom row shows similarity space plots of the temporal lobe seed voxels according to the similarity of their connectivity profiles. The colouring is a ‘ranked’ RGB representation of a voxel's location in the connective similarity space. The top row displays the results projected onto the temporal lobes, with a given voxel's RGB colour retained as it moves between similarity and anatomic spaces Note that the colouring is ranked in order to improve visualisation; see supplementary animation for the raw colours and a depiction of the mapping between spaces.

**Fig. 2:**
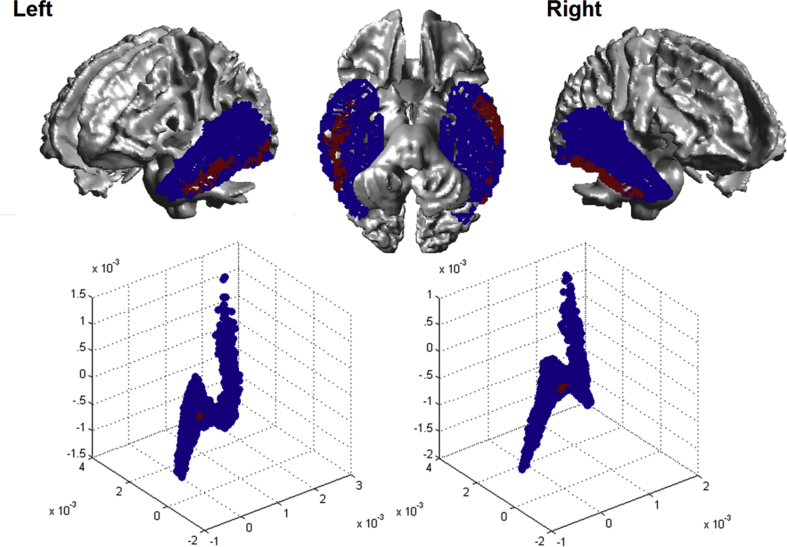
Left and right hemispheric connectivity space highlighting regions around the centre of mass in red. This region in the centre of the connectivity space is the area of least difference, on average, to all other areas.

**Fig. 3:**
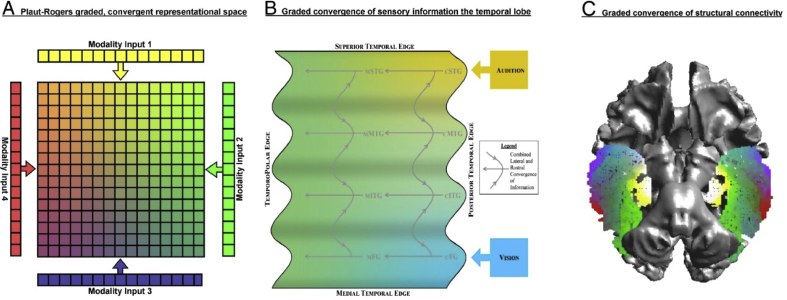
The graded convergence of information A) as proposed by the (Plaut, 2002) model; B) proposed convergence based on white matter connections by (Binney et al., 2012). C) the results of the current Laplacian eigenmap showing the convergence of connectivity in the ventral ATL. Panels A and B were originally published in the journal of cognitive neuroscience (Binney et al., 2012); reproduced with permission.

### 2.5 Data and code sharing

The ethics application for this study was such that did not cater for the public archiving of MRI data. Hence, participants did not provide sufficient informed consent for such public archiving. However, researchers who would like to access the raw data should contact the corresponding author who will liase with the ethics committee that approved the study. Where compatible with the General Data Protection Regulation (GDPR) and in line with ethics decisions made by the committee, as much data that is necessary to reproduce the results will be released to the individual researcher.

Code used for this project, with some modifications for readability and removal of any system links, has been made available for review on the Open Science Framework (OSF). Furthermore, pre-processed data along with accompanying code has been provided to replicate the results found in this paper (https://osf.io/gsve9/). No part of the study procedures or analyses was pre-registered in a time-stamped, institutional registry prior to the research being conducted.

Parts of the methods (specifically the PICo tractography code) are covered by patents held by Bioxydyn and therefore source code has not been released and any commercial use is forbidden unless express permission is given by the company. The code used to perform the CSD contains commercially sensitive information belonging to Dr Hamied Haroon which cannot be publicly released.

## 3 Results

The generalised eigendecomposition of the Laplacian of the similarity matrix produced only one zero eigenvalue in both hemispheres, hence the similarity ‘graphs’ were fully connected. The second (the algebraic connectivity), third and fourth eigenvalues were .63, .77 and .88 respectively on the left and .64, .80 and .88 respectively on the right. The largest eigenvalue was 1 bilaterally. This suggests that the structural connectivity of the temporal lobe does not separate into clearly defined clusters but that there are graded differences in connectivity. The connectivity showed qualitatively similar patterns in both hemispheres.

The rows of the first three non-zero eigenvectors were taken to be coordinates of points in a three dimensional ‘connective similarity’ space (ℝ^3^) and their equivalent feature scaled values taken to be coordinate values on the RGB colour space (as described above). Fig. 1 shows the results of the analysis plotted on the brain and in the connective similarity space. The voxels within the similarity space are plotted according to how similar the temporal seed voxels' connectivity profiles were to each other, with voxels demonstrating greater similarity positioned closer together. One can consider the temporal lobe being warped from a space where voxels are plotted according to their ‘anatomic’ similarity to a space where they are plotted according to their ‘connective’ similarity and vice-versa. An animation depicting the morphing between the anatomic space to the similarity space can be found in the supplementary materials.

As can be seen from Fig. 1, the hippocampus (coloured white to yellow) is the most distinctly connected region within the temporal lobe, while the lateral areas demonstrate a graded transition of connectivity. Indeed, while the hippocampus forms the distant white ‘tail’ in the similarity space, the lateral temporal lobe forms the quasi-triangular green, red and purple ‘head’. While the more primary visual and auditory cortices lie on the edges of the triangular head (red-purple), the anterior temporal lobe map to the centre of the space, indicating that it has gradedly similar connectivity with all of the primary temporal areas. Since the colours of the centre of the space are hard to visually distinguish, voxels within the 10th percentile of Euclidian distance from the centre of mass were identified (see red region in Fig. 2). This identified a ventral anterior temporal region along the more lateral surface. These correspond to the temporal seed voxels with the least ‘distinct’ connectivity profiles, demonstrating a connectivity profile similar to other temporal regions, and representing an area of ‘convergent’ similarity.

## 4 Discussion

The current study used a data driven approach to examine the connective organisation of the temporal lobe, particularly in relation to variations across the lateral surface involving the ventral ATL. The first main finding is that there is a clear distinction between the patterns of connectivity between medial temporal regions (specifically the hippocampal region), and the lateral regions. While the medial temporal lobe demonstrated a very distinctive connectivity profile compared to the rest of the temporal cortex, the lateral temporal lobe was associated with more graded similarity and transitions between regions. This is not surprising given the functional and cytoarchitectonic differences (allocortex vs. neocortex) of the medial temporal lobe (Posimo, Titler, Choi, Unnithan, & Leak, 2013). Indeed, Nestor et al. (2006) describe dissociations between the impairments in semantic dementia (semantic impairment without amnesia following ventrolateral centred atrophy) versus early Alzheimer's disease (amnesia without semantic impairment following ventromedial pathology). These findings align with the major connectivity distinction found within this study. As such follow-up work using similar methods using patient groups would be welcome.

The second key finding of the current study was that along the graded lateral region, a zone of convergence of connectivity could be delineated, specifically involving the ventral ATL. The connectivity patterns within this zone of convergence demonstrated similarity with both the primary and secondary visual and auditory cortices, highlighting a region where these different modalities of information may become integrated. Finally, we note that there were little qualitative interhemispheric differences in connectivity. This seemingly contradicts previous work that has observed interhemispheric differences in strength of the temporal lobe's connectivity to other structures (Papinutto et al., 2016). These facts do not necessarily conflict due to the nature of the different approaches. First, the current work does not focus on connectivity strength but on the similarity between the structural connectivity patterns of temporal lobe voxels to the rest of the brain; hence while connectivity strength may change, the spatial pattern of the network may remain the same. Second, while there aren't great differences between the two hemispheres in our study there are some minor difference in the connectivity space in both hemispheres

Rogers et al. (Rogers et al., 2004) and Patterson et al. (Patterson et al., 2007) were the first to suggest that the ATL acts as a multi-modal hub involved in integrating modality-specific semantic information from their respective distributed cortical regions (or ‘spoke’ nodes). The hub-and-spoke model enables modality-specific nodes within the network to ‘communicate’ with one another via the hub node. As such, the hub acts as an integrator of the different features of an object that are processed at the spokes and allows hub-and-spokes to compute the variable and arbitrary mappings of features into coherent generalizable concepts (cf. Lambon Ralph, Sage, Jones, & Mayberry, 2010). This is in contrast with a ‘distributed-only’ model of semantics where integration occurs as an emergent property across the whole network (Martin, 2007); or with a putative ‘hub-only’ idea of semantics where, in a neo-phrenological fashion, the ATL is ‘the region’ for semantic cognition (Lambon Ralph, 2014). Indeed, there is an ever increasing body of evidence supporting the conception that the ATL plays a crucial role in multi-modal semantics, including neurological conditions such as semantic dementia (Hodges et al., 1992), patients who have undergone ATL resection for medically intractable epilepsy (Lambon Ralph et al., 2012, Rice et al., 2018), sub-dural cortical electrode investigations (Shimotake et al., 2015), fMRI in healthy participants (Jackson et al., 2016, Jefferies, 2013) and transient ‘lesioning’ methods such as transcranial magnetic stimulation (Lambon Ralph et al., 2009, Pobric et al., 2007).

In response to the growing body of research on semantic cognition, the hub-and-spoke model has become more nuanced. The single hub has been adapted into a graded hub along the ventral anterior temporal lobe (Binney et al., 2012, Rice et al., 2015, Visser et al., 2010). Within this framework, as information travels from the primary cortices, through association cortices and towards the ATL, the concepts become less modality specific in a graded fashion (see Fig. 3) (Binney et al., 2012, Lambon Ralph, 2014, Rice et al., 2015). The idea of a graded convergence of representations was first proposed by (Plaut, 2002) in his computational model of semantics (see Fig. 3A). This model was developed to reconcile theories that proposed the existence of extreme ends of functional specialisation within the semantic system (Lambon Ralph et al., 2017).

Previous studies examining the structural connectivity of the temporal region have also suggested an underlying convergence and gradation of multi-modal information within the temporal lobe in support of the (Plaut, 2002) model, although not formally investigated [(Binney et al., 2012) Fig. 3B]. The current data driven examination of the connectivity gradients within the temporal cortex provide further support for this graded convergence model of semantics (Fig. 3C).

The current results identified the ventral portion of the ATL as the zone with the highest connective convergence making it, together with evidence from patient data (Hoffman, Evans, & Lambon Ralph, 2014), a prime candidate brain region for the most multi-modal computations. Indeed, when one highlights the zone of convergence (red region in Fig. 2) and compares it to previous work (Binder et al., 2009, Visser et al., 2012), the zone overlaps with the anterior portion of the temporal semantic network. Furthermore, this area has been shown to activate in response to more than one modality (Fig. 3B), reflecting the finding of (Visser et al., 2012) that both the middle temporal gyrus and the ventral anterior temporal lobe are crucial for multi-modal semantics. As such, the current evidence aligns with recent conceptualisations of the hub-and-spoke model which posit that multi-modal integration does not occur in an all-or-none fashion solely within the putative ‘hub’ region, but involves the gradual convergence of modality-specific information along the course of the semantic network to become completely multi-modal (or indeed, amodal) within the ventral ATL hub (Lambon Ralph et al., 2017).

The current study employed a data-driven approach to examine the convergence of connectivity within the temporal lobe, utilising white matter tractography and Laplacian eigenmapping. These methods have several key strengths, as well as important limitations which need to be taken into consideration in interpreting the current results.

*Tractography* infers the structure and course of white matter connectivity indirectly via the diffusion of water within the brain. As such, it is subject to the generation of a degree of ‘false-positive’ and ‘false-negative’ fibres. These connective inaccuracies are principally a problem if one is particularly interested in the precise delineation of corticocortical connectivity. However, the current study was not focused on the delineation of such connections, but rather on the patterns of connective similarity and difference across cortical voxels, which is more robust to small inaccuracies in the connective data (Johansen-Berg et al., 2004). As such, while false negative and positive streamlines may affect the absolute precision of our similarity metrics, the overall impact of these inaccuracies is reduced in comparison to traditional white matter tractography mapping.

*Eigenmapping*, in the context used for this investigation, is a technique that allows the spectral transformation of voxels in a ‘connectivity’ space to be mapped onto anatomy. The three primary advantages accorded by this approach are first, the ability to visualise the closeness of voxels in the connective similarity space. One can examine this space and visually identify whether hard clustering of the data makes sense. Indeed, the spectral transformation represents the initial data transformation that is used prior to the clustering portion of spectral clustering algorithms (Von Luxburg, 2007). In the case of these data, one can clearly see that hard clustering the connectivity space may create a false sense of ‘distinctness’ of brain areas within the temporal lobe rather than the gradations what are currently clear.

Secondly, the current approach does not cluster voxels into distinct groups, instead focusing on the pattern of change in voxels connectivity profiles. Indeed, avoiding clustering also allows the investigation of the area that is maximally similar to all other areas; a location that by its very nature would not cluster well.

Finally, in contrast to spectral reordering, or an eigenmapping based only on the first non-zero eigenvector, our approach uses the RGB space in order to increase the dimensionality of the visualisation in order to gain added information to those approaches that only use a single dimension (Johansen-Berg et al., 2004, Margulies et al., 2016).

In summary, previous research has indicated that the ventral ATL is an essential area in processing multi-modal semantic information. It is thought to be a graded hub that integrates modality specific information from its spokes (Patterson et al., 2007, Rice et al., 2015). It has also been shown that the ventral ATL receives long range connections from distant primary sensory regions and there is also a zone that has particularly strong local connectivity (Binney et al., 2012). This work has shown that the connectivity profiles within the temporal cortex do indeed converge onto the ventral ATL and consistent with the proposition that the ventral ATL is a zone of convergence in the brain; a graded semantic hub.

## Author contributions

CJB, LLC and MALR conceived the study. CJB and NJTB ran the analysis. GJMP and NJTB advised on technical matters. All authors were involved in reviewing the manuscript.

## Competing interests

GJMP is a shareholder and director of Bioxydyn, a company with an interest in imaging services and products.

## Figures and Tables

**figs1:**
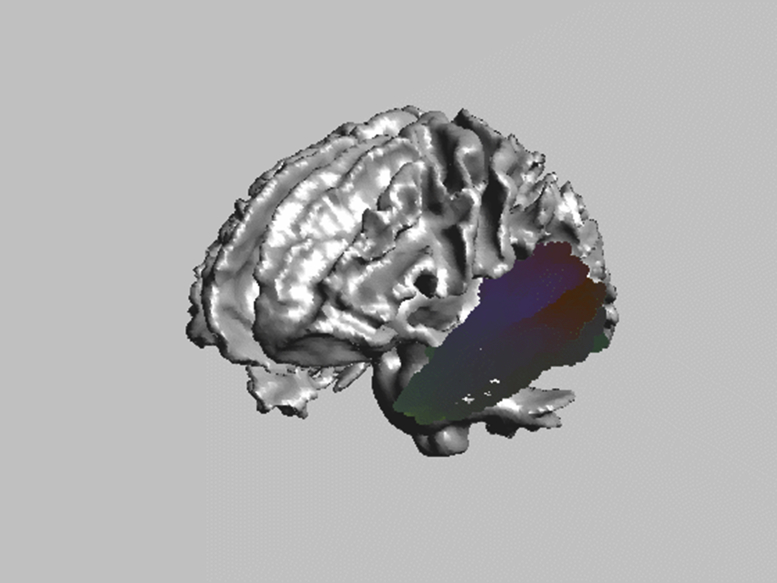


## References

[bib1] Abel T.J., Rhone A.E., Nourski K.V., Kawasaki H., Oya H., Griffiths T.D. (2015). Direct physiologic evidence of a heteromodal convergence region for proper naming in human left anterior temporal lobe. The Journal of Neuroscience.

[bib2] Ashburner J. (2007). A fast diffeomorphic image registration algorithm. Neuroimage.

[bib3] Bajada C.J., Jackson R.L., Haroon H.A., Azadbakht H., Parker G.J.M., Lambon Ralph M.A. (2017). A graded tractographic parcellation of the temporal lobe. Neuroimage.

[bib4] Barnard Stephen T., Pothen A., Simon H. (1995). A spectral algorithm for envelope reduction of sparse matrices. Numerical Linear Algebra with Applications.

[bib5] Barnard S.T., Pothen A., Simon H.D. (1993). A spectral algorithm for envelope reduction of sparse matrices. Supercomputing.

[bib6] Belkin M., Niyogi P. (2002). Laplacian eigenmaps and spectral techniques for embedding and clustering. Advances in Neural Information Processing Systems.

[bib7] Belkin M., Niyogi P. (2003). Laplacian eigenmaps for dimensionality reduction and data representation. Neural Computation.

[bib8] Binder J.R., Desai R.H., Graves W.W., Conant L.L. (2009). Where is the semantic system? A critical review and meta-analysis of 120 functional neuroimaging studies. Cerebral Cortex.

[bib9] Binney R.J., Embleton K.V., Jefferies E., Parker G.J.M., Ralph M.A.L. (2010). The ventral and inferolateral aspects of the anterior temporal lobe are crucial in semantic memory: Evidence from a novel direct comparison of distortion-corrected fMRI, rTMS, and semantic dementia. Cerebral Cortex.

[bib10] Binney R.J., Parker G.J.M., Lambon Ralph M.A. (2012). Convergent connectivity and graded specialization in the rostral human temporal lobe as revealed by diffusion-weighted imaging probabilistic tractography. Journal of Cognitive Neuroscience.

[bib11] Bozeat S., Lambon Ralph M.A., Patterson K., Garrard P., Hodges J.R. (2000). Non-verbal semantic impairment in semantic dementia. Neuropsychologia.

[bib12] Cerliani L., Thomas R.M., Jbabdi S., Siero J.C.W., Nanetti L., Crippa A. (2012). Probabilistic tractography recovers a rostrocaudal trajectory of connectivity variability in the human insular cortex. Human Brain Mapping.

[bib13] Chen L., Lambon Ralph M.A., Rogers T.T. (2017). A unified model of human semantic knowledge and its disorders. Nature Human Behaviour.

[bib14] Craddock R.C., James G.A., Holtzheimer P.E., Hu X.P., Mayberg H.S. (2012). A whole brain fMRI atlas generated via spatially constrained spectral clustering. Human Brain Mapping.

[bib15] Devlin J.T., Sillery E.L., Hall D.A., Hobden P., Behrens T.E.J., Nunes R.G. (2006). Reliable identification of the auditory thalamus using multi-modal structural analyses. Neuroimage.

[bib16] Ding J., Chen K., Chen Y., Fang Y., Yang Q., Lv Y. (2016). The left fusiform gyrus is a critical region contributing to the core behavioral profile of semantic dementia. Frontiers in Human Neuroscience.

[bib17] Eickhoff S.B., Thirion B., Varoquaux G., Bzdok D. (2015). Connectivity-based parcellation: Critique and implications. Human Brain Mapping.

[bib18] Embleton K.V., Haroon H.A., Morris D.M., Ralph M.A.L., Parker G.J.M. (2010). Distortion correction for diffusion-weighted MRI tractography and fMRI in the temporal lobes. Human Brain Mapping.

[bib19] Fiedler M. (1973). Algebraic connectivity of graphs. Czechoslovak Mathematical Journal.

[bib20] Galton C.J., Patterson K., Graham K., Lambon-Ralph M.A., Williams G., Antoun N. (2001). Differing patterns of temporal atrophy in Alzheimer's disease and semantic dementia. Neurology.

[bib21] Guell X., Schmahmann J.D., Gabrieli J.D., Ghosh S.S. (2018). Functional gradients of the cerebellum. eLife.

[bib22] Haak K.V., Marquand A.F., Beckmann C.F. (2016). Connectopic mapping with resting-state fMRI.

[bib23] Haroon H.A., Morris D.M., Embleton K.V., Alexander D.C., Parker G.J.M. (2009). Using the model-based residual bootstrap to quantify uncertainty in fiber orientations from Q-ball analysis. IEEE Transactions on Medical Imaging.

[bib24] Haroon H.A., Morris D.M., Embleton K.V., Parker G.J. (2009). Model based residual bootstrap of constrained spherical deconvolution for probabilistic segmentation and tractography. Presented at the proceedings of the 17th scientific meeting and exhibition of the international society for magnetic resonance in medicine.

[bib25] Hodges J.R., Patterson K., Oxbury S., Funnell E. (1992). Semantic dementia. Brain.

[bib26] Hoffman P., Evans G.A.L., Lambon Ralph M.A. (2014). The anterior temporal lobes are critically involved in acquiring new conceptual knowledge: Evidence for impaired feature integration in semantic dementia. Cortex.

[bib27] Jackson R.L., Bajada C.J., Rice G.E., Cloutman L.L., Lambon Ralph M.A. (2017). An emergent functional parcellation of the temporal cortex. Neuroimage.

[bib28] Jackson R.L., Hoffman P., Pobric G., Lambon Ralph M.A. (2016). The semantic network at work and rest: Differential connectivity of anterior temporal lobe subregions. The Journal of Neuroscience.

[bib29] Jefferies E. (2013). The neural basis of semantic cognition: Converging evidence from neuropsychology, neuroimaging and TMS. Cortex.

[bib30] Jeurissen B., Leemans A., Jones D.K., Tournier J.-D., Sijbers J. (2011). Probabilistic fiber tracking using the residual bootstrap with constrained spherical deconvolution. Human Brain Mapping.

[bib31] Johansen-Berg H., Behrens T.E.J., Robson M.D., Drobnjak I., Rushworth M.F.S., Brady J.M. (2004). Changes in connectivity profiles define functionally distinct regions in human medial frontal cortex. Proceedings of the National Academy of Sciences of the United States of America.

[bib32] Karolis V., Corbetta M., Thiebaut de Schotten M. (2018). Architecture of functional lateralisation in the human brain. BioRxiv.

[bib33] Lambon Ralph M.A. (2014). Neurocognitive insights on conceptual knowledge and its breakdown. Philosophical Transactions of the Royal Society of London. Series B, Biological Sciences.

[bib34] Lambon Ralph M.A., Ehsan S., Baker G.A., Rogers T.T. (2012). Semantic memory is impaired in patients with unilateral anterior temporal lobe resection for temporal lobe epilepsy. Brain: a Journal of Neurology.

[bib35] Lambon Ralph M.A., Jefferies E., Patterson K., Rogers T.T. (2017). The neural and computational bases of semantic cognition. Nature Reviews. Neuroscience.

[bib36] Lambon Ralph M.A., Pobric G., Jefferies E. (2009). Conceptual knowledge is underpinned by the temporal pole bilaterally: Convergent evidence from rTMS. Cerebral Cortex.

[bib0070] Lambon Ralph M.A., Sage K., Jones R.W., Mayberry E.J. (2010). Coherent concepts are computed in the anterior temporal lobes. Proceedings of the National Academy of Sciences.

[bib37] Margulies D.S., Ghosh S.S., Goulas A., Falkiewicz M., Huntenburg J.M., Langs G. (2016). Situating the default-mode network along a principal gradient of macroscale cortical organization. Proceedings of the National Academy of Sciences of the United States of America.

[bib38] Martin A. (2007). The representation of object concepts in the brain. Annual Review of Psychology.

[bib39] Mazziotta J., Toga A., Evans A., Fox P., Lancaster J., Zilles K. (2001). A probabilistic atlas and reference system for the human brain: International Consortium for Brain Mapping (ICBM). Philosophical Transactions of the Royal Society of London. Series B, Biological Sciences.

[bib40] Mion M., Patterson K., Acosta-Cabronero J., Pengas G., Izquierdo-Garcia D., Hong Y.T. (2010). What the left and right anterior fusiform gyri tell us about semantic memory. Brain: a Journal of Neurology.

[bib41] Mummery C.J., Patterson K., Price C.J., Ashburner J., Frackowiak R.S., Hodges J.R. (2000). A voxel-based morphometry study of semantic dementia: Relationship between temporal lobe atrophy and semantic memory. Annals of Neurology.

[bib42] Nestor P.J., Fryer T.D., Hodges J.R. (2006). Declarative memory impairments in Alzheimer's disease and semantic dementia. Neuroimage.

[bib43] Ng A.Y., Jordan M.I., Weiss Y. (2002). On spectral clustering: Analysis and an algorithm. Advances in Neural Information Processing Systems.

[bib44] Noonan K.A., Jefferies E., Visser M., Lambon Ralph M.A. (2013). Going beyond inferior prefrontal involvement in semantic control: Evidence for the additional contribution of dorsal angular gyrus and posterior middle temporal cortex. Journal of Cognitive Neuroscience.

[bib45] Oldfield R.C. (1971). The assessment and analysis of handedness: The Edinburgh inventory. Neuropsychologia.

[bib46] Papinutto N., Galantucci S., Mandelli M.L., Gesierich B., Jovicich J., Caverzasi E. (2016). Structural connectivity of the human anterior temporal lobe: A diffusion magnetic resonance imaging study. Human Brain Mapping.

[bib47] Parker G.J.M., Alexander D.C. (2005). Probabilistic anatomical connectivity derived from the microscopic persistent angular structure of cerebral tissue. Philosophical Transactions of the Royal Society of London. Series B, Biological Sciences.

[bib48] Parker G.J.M., Haroon H.A., Wheeler-Kingshott C.A.M. (2003). A framework for a streamline-based probabilistic index of connectivity (PICo) using a structural interpretation of MRI diffusion measurements. Journal of Magnetic Resonance Imaging: JMRI.

[bib49] Patterson K., Nestor P.J., Rogers T.T. (2007). Where do you know what you know? The representation of semantic knowledge in the human brain. Nature Reviews. Neuroscience.

[bib50] Plaut D.C. (2002). Graded modality-specific specialisation in semantics: A computational account of optic aphasia. Cognitive Neuropsychology.

[bib51] Pobric G., Jefferies E., Ralph M.A.L. (2007). Anterior temporal lobes mediate semantic representation: Mimicking semantic dementia by using rTMS in normal participants. Proceedings of the National Academy of Sciences of the United States of America.

[bib52] Posimo J.M., Titler A.M., Choi H.J.H., Unnithan A.S., Leak R.K. (2013). Neocortex and allocortex respond differentially to cellular stress in vitro and aging in vivo. Plos One.

[bib53] Rice G.E., Caswell H., Moore P., Hoffman P., Lambon Ralph M.A. (2018). The roles of left versus right anterior temporal lobes in semantic memory: A neuropsychological comparison of postsurgical temporal lobe epilepsy patients. Cerebral Cortex.

[bib54] Rice G.E., Hoffman P., Lambon Ralph M.A. (2015). Graded specialization within and between the anterior temporal lobes. Annals of the New York Academy of Sciences.

[bib55] Rice G.E., Lambon Ralph M.A., Hoffman P. (2015). The roles of left versus right anterior temporal lobes in conceptual knowledge: An ALE meta-analysis of 97 functional neuroimaging studies. Cerebral Cortex.

[bib56] Rogers T.T., Lambon Ralph M.A., Garrard P., Bozeat S., McClelland J.L., Hodges J.R. (2004). Structure and deterioration of semantic memory: A neuropsychological and computational investigation. Psychological Review.

[bib57] Scoville W.B., Milner B. (1957). Loss of recent memory after bilateral hippocampal lesions. Journal of Neurology, Neurosurgery, and Psychiatry.

[bib58] Shi J., Malik J. (1997). Normalized cuts and image segmentation. Proceedings of IEEE computer society conference on computer vision and pattern recognition.

[bib59] Shi J., Malik J. (2000). Normalized cuts and image segmentation. IEEE Transactions on Pattern Analysis and Machine Intelligence.

[bib60] Shimotake A., Matsumoto R., Ueno T., Kunieda T., Saito S., Hoffman P. (2015). Direct exploration of the role of the ventral anterior temporal lobe in semantic memory: Cortical stimulation and local field potential evidence from subdural grid electrodes. Cerebral Cortex.

[bib61] The MathWorks I. (2012). MATLAB and statistics toolbox [7.14.0.739 (R2012a)].

[bib62] Tournier J.-D., Calamante F., Connelly A. (2007). Robust determination of the fibre orientation distribution in diffusion MRI: Non-negativity constrained super-resolved spherical deconvolution. Neuroimage.

[bib63] Visser M., Jefferies E., Embleton K.V., Lambon Ralph M.A. (2012). Both the middle temporal gyrus and the ventral anterior temporal area are crucial for multimodal semantic processing: Distortion-corrected fMRI evidence for a double gradient of information convergence in the temporal lobes. Journal of Cognitive Neuroscience.

[bib64] Visser M., Jefferies E., Lambon Ralph M.A. (2010). Semantic processing in the anterior temporal lobes: A meta-analysis of the functional neuroimaging literature. Journal of Cognitive Neuroscience.

[bib65] Von Luxburg U. (2007). A tutorial on spectral clustering. Statistics and Computing.

